# Substitution of marijuana for opioids in a national survey of US adults

**DOI:** 10.1371/journal.pone.0222577

**Published:** 2019-10-04

**Authors:** Julie H. Ishida, Peggy O. Wong, Beth E. Cohen, Marzieh Vali, Stacey Steigerwald, Salomeh Keyhani

**Affiliations:** 1 Department of Medicine, Division of Nephrology, San Francisco VA Medical Center, San Francisco, California, United States of America; 2 Department of General Internal Medicine, San Francisco VA Medical Center, San Francisco, California, United States of America; Medical University Graz, AUSTRIA

## Abstract

Opioid prescriptions for chronic pain and subsequent opioid-related complications have risen dramatically in the US. Recent data suggest that medical marijuana laws have been associated with lower state-level opioid overdose mortality. In a national survey, we examined the prevalence of substitution of marijuana for opioids among US adults taking opioids for pain.Using GfK’s KnowledgePanel, we conducted an Internet-based survey of a nationally representative sample of 16,280 adults in 2017 about individual perceptions and use of marijuana. We developed questions designed to assess the extent and reasons for substitution of marijuana for opioids. We examined opioid substitution among respondents with a history of ever using marijuana who used opioids in the past 12 months. There were 9,003 respondents, corresponding to a 55.3% response rate. The mean age was 48 years. Among the 5% (n = 486) who reported ever using marijuana and using opioids in the past year, 43% used opioids daily, and 23% reported current (past 30 day) marijuana use. Forty-one percent reported a decrease or cessation of opioid use due to marijuana use; 46% reported no change in opioid use; and 8% reported an increase in opioid use. We found that a substantial number of US adults reported that they substituted marijuana for opioids.

## Introduction

Chronic pain affects approximately one-third of the U.S. population, and opioid prescriptions have substantially increased over the last 20 years [1]. In parallel, there has been an increase in opioid-related complications, with opioid overdose deaths quadrupling between 1999 and 2015 [2]. Growing concerns about the risks of opioids, including overdose-related deaths and opioid use disorder, have prompted greater focus on the more judicious use of these agents for managing pain and the need to identify other agents to treat pain [3].

The data on the efficacy of cannabinoids in the management of pain is evolving. In a systematic review, there was low-strength evidence that cannabis is effective for treating neuropathic pain and insufficient evidence of its effectiveness for other types of pain [4]. The American Academy of Neurology has endorsed use of cannabinoids for the pain and spasticity associated with multiple sclerosis but cautions that the safety profile of cannabinoids has not been compared to other approved drugs [5]. Despite the lack of robust evidence for efficacy of cannabinoids in pain management, marijuana has been approved by legislatures or ballot initiative for the management of pain in over 30 states [6].

Recent data suggest that medical marijuana laws have been associated with lower state-level opioid overdose mortality, hospitalizations related to opioid complications, detection of opioids among fatally injured drivers, and prescription of analgesics [7]. These ecologic studies, while hypothesis generating, do not inform our understanding of the individual effects of marijuana use or combined marijuana and opioid use. Prospective cohort studies and clinical trials are needed to improve our understanding of the effects of cannabis on pain management. Nonetheless, these studies have spurred discussion about the potential for marijuana to serve as a substitute for opioids, particularly in contexts where marijuana is increasingly available through legalization. Small surveys of convenience samples of American and Canadian marijuana users have reported that substitution of marijuana for opioids is common, ranging from approximately 30% to 97% [8, 9]. To our knowledge, there are no nationally representative surveys examining substitution and reasons for substitution among the general US adult population. We examined the prevalence and reasons for substitution of marijuana for opioids among US adults taking opioids for pain, as well as the factors associated with substitution.

## Methods

### Survey development

Details of survey development have been previously published [10]. The survey questions were designed based on a review of the literature and existing national surveys and interviews with substance abuse experts and marijuana distributors and dispensary staff [10]. The survey asks about a wide range of topics, including perception of risks and benefits associated with marijuana use, comparisons of marijuana to other substances (tobacco, alcohol), and pertinent public health questions relevant to implementing marijuana legalization. The current study is based on the questions that were designed to assess the extent and reasons for substitution of marijuana for opioids. All questions used Likert scales for response options and were edited to meet an 8^th^-grade reading level. Prior to administration, our survey was tested on a convenience sample of 40 adults to ensure question reliability and validity. Volunteers were comprised of a panel of patients from the investigator’s (SK, BC) clinics and were offered no incentives to volunteer (survey available in S1 File).

We ascertained opioid use (using the colloquial term “opiate”) with the following question: “In the past 12 months, have you regularly taken opiate medications such as Vicodin, Percocet, or OxyContin to treat pain? Do not include pain medications that can be bought without a prescription such as aspirin, Tylenol, or Advil.” We ascertained marijuana use with the following questions: “Have you ever used marijuana?” and “How long has it been since you last used marijuana?”

We ascertained substitution of marijuana for opioids as follows: “Have you noticed a change in the amount of opiate medications you need or use for pain because of your marijuana use?” Response options were a) “Yes, I need a lot more opiate medication,” b) “Yes, I need slightly more opiate medication,” c) “No change,” d) “Yes, I need slightly less opiate medication,” e) “Yes, I need a lot less opiate medication,” and f) “I have been able to stop using opiate medications.” Among those endorsing substitution, we also asked about reasons for substitution. Response options were a) “Better pain management with marijuana,” b) “Fewer side effects from marijuana,” c) “Fewer withdrawal symptoms with marijuana,” d) “Marijuana is easier to obtain,” e) “Marijuana is cheaper,” and f) “More social acceptance from marijuana use.” Multiple selections were allowed for these follow-up questions.

### Sampling strategy

In 2017, we conducted an Internet-based survey of 16,280 adults about perceptions of marijuana using KnowledgePanel (GfK Custom Research North America), a nationally representative panel of the civilian, noninstitutionalized US population (aged ≥18 years) [10]. KnowledgePanel has been in use for surveying public opinion since 1999 [11–14]. GfK created a representative sample of US adults by random sampling of addresses [15]. The address-based sampling (ABS) covers 97% of the country and encompasses a statistical representation of the US population. Adults were invited to join through mailings, postcards, and follow up letters. Nonresponding households were called. Participation included: completing and mailing back the paper invitation; calling a toll-free number provided by GfK; and completing a recruitment form online [15]. All participants receive the survey in the same manner, households without Internet access are provided with an Internet connection and a tablet to ensure participation. All participants in the panel are sampled with a known probability of selection. No one can volunteer to participate. Participants do not receive monetary incentives to participate but receive points that can be used towards purchases. Participants are provided with no more than six surveys a month and are expected to complete an average of four surveys a month. (Further details on the sampling strategy of GfK’s KnowledgePanel is provided here: (https://www.gfk.com/fileadmin/user_upload/dyna_content/US/documents/KnowledgePanel_Methodology.pdf). For the purposes of future investigation into the role of marijuana legalization on use, California residents and young adults aged 18 to 26 years old were oversampled. Sampling weights were provided by GfK.

### Survey administration

The survey was launched on September 27, 2017 to a total of 16,280 US adults 18 years and older and was completed on October 9, 2017. The survey was administered using an online format. This study was considered exempt from review by the Committee on Human Subject Research, University of California, San Francisco.

### Statistical analysis

Our response rate, defined as the ratio of all respondents to all potential respondents, was determined using methodology as outlined by the American Association for Public Opinion Research [16]. Characteristics of the survey respondents were weighted using weights provided by GfK to approximate the US population based on age, sex, race, ethnicity, education, household income, home ownership and metropolitan area. All analyses used weighting commands using the weight variable provided by GfK to generate national estimates. To determine how well our sample compared to a national federally-sponsored survey on substance abuse and marijuana use, we first compared the socio-demographic characteristics of our survey respondents to those of the National Survey on Drug Use and Health (NSDUH) [17]. NSDUH is an annual federal survey implemented by the Substance Abuse and Mental Health Services Administration (SAMHSA), which is an agency of the Department of Health and Human Services (DHHS). NSDUH provides data on substance abuse epidemiology in the US [17]. We then examined opioid substitution among respondents with a history of ever using marijuana who used opioids in the past 12 months. We used logistic regression to determine associations between socio-demographic characteristics (e.g., age, gender, race/ethnicity, education, household income, employment) and status of marijuana legalization in the state of residence and substitution of marijuana for opioids. The cases who were categorized as “ever” marijuana users with opioid use within the past 12 months who refused to answer were excluded from this logistic model. Analyses were conducted using R statistical software (version R-3.4.0). There were very few participants with missing data (n = 4) and these cases were dropped from the analysis. This study was considered exempt by the University of California, San Francisco Committee on Human Research.

## Results

There were 9,003 respondents, corresponding to a 55.3% response rate. Baseline characteristics of respondents were similar to respondents from the National Survey on Drug Abuse and Health, though our respondents had a slightly higher average income, (**Table A in S1 File**) suggesting our sample was representative of the US population [10]. The mean age was 48 years, 48% were male, 64% were white, and 64% lived in a state in which marijuana was legal. Among this national sample, forty-six percent reported ever using marijuana, and 8% reported regular use of opioids for pain in the past year.

Among the 5% (n = 486) who reported ever using marijuana and using opioids in the past year, 43% used opioids daily, and 23% reported current (past 30 day) marijuana use (**Table 1**). Forty-one percent reported a decrease or cessation of opioid use due to marijuana use; 46% reported no change in opioid use; and 8% reported an increase in opioid use. The most commonly reported reasons for substitution were better pain management (36%) and fewer side effects (32%) and withdrawal symptoms (26%), compared to the non-medical reasons for use: cheaper (13%) and more social acceptance from marijuana use (13%). In multivariable analyses, we found no association between socio-demographics or status of marijuana legalization in the state of residence and substitution (**Table 2**).

**Table 1:** Frequency of opioid and marijuana use, prevalence of substitution of marijuana for opioids, and reasons for substitution among persons reporting marijuana and opioid use in a national survey of US adults conducted in fall 2017.

Characteristic	Ever Marijuana Users with Opioid Use with the Past 12 Months (n = 486) n (%)*
***Frequency of Opioid Use ***	
Daily	197 (43)
Weekly	95 (18)
Monthly	42 (9)
Less than monthly	150 (29)
Refused	2 (0)
***Frequency of Marijuana Use***	
Current (within the past 30 days)	113 (23)
Past year (more than 30 days but within the past 12 months)	80 (15)
More than past year	293 (62)
***Change in Opioid Requirement due to Marijuana Use ***	
A lot more opioid needed	14 (4)
Slightly more opioid needed	18 (4)
No change in opioid use	244 (46)
Slightly less opioid needed	31 (8)
A lot less opioid needed	63 (13)
Stopped opioid use	93 (20)
Refused	23 (5)
***Reasons for Decrease or Cessation of Opioid Use***	
Better pain management with marijuana	71 (36)
Fewer side effects from marijuana	63 (32)
Fewer withdrawal symptoms with marijuana	40 (26)
Marijuana is easier to obtain	22 (16)
Marijuana is cheaper	17 (13)
More social acceptance from marijuana use	20 (13)
Other	72 (36)
Refused	4 (4)

*Numbers are unweighted, and percentages are weighted to approximate the US population. We used weights provided by GfK to approximate the US population based on socio-demographic factors (e.g., age, gender, race, ethnicity, education, household income, home ownership, and metropolitan area).

**Table 2:** Characteristics associated with substitution of marijuana for opioids.

Characteristic	Ever Marijuana Users with Opioid Use with the Past 12 Months (n = 486) n (%)*	Did Substitute Marijuana (n = 187) n (%)*	Did Not Substitute Marijuana (n = 276) n (%)*	OR (95% CI)
***Age (years)***				
18–34	66 (14)	32 (51)	31 (49)	1.86 (0.91, 3.79)
35–49	115 (24)	42 (39)	66 (61)	1.03 (0.56, 1.92)
50–64	198 (41)	75 (39)	115 (61)	1.08 (0.63, 1.83)
≥65	107 (22)	38 (37)	64 (63)	1.00 (ref)
***Gender***				
Female	227 (47)	82 (39)	130 (61)	0.81 (0.55, 1.20)
Male	259 (53)	105 (42)	146 (58)	1.00 (ref)
***Race/Ethnicity***				
Black/Non-Hispanic	47 (10)	18 (40)	27 (60)	1.03 (0.52, 2.02)
Hispanic	50 (10)	23 (48)	25 (52)	1.33 (0.70, 2.53)
Other/Non-Hispanic	31 (6)	13 (45)	16 (55)	1.30 (0.59, 2.84)
White/Non-Hispanic	358 (74)	133 (39)	208 (61)	1.00 (ref)
***Education***				
High school or less	172 (35)	71 (45)	87 (55)	1.38 (0.79, 2.40)
Some college	179 (37)	68 (39)	106 (61)	1.12 (0.69, 1.84)
Bachelor's degree or higher	135 (28)	48 (37)	83 (63)	1.00 (ref)
***Household Income***				
<$20,000	114 (23)	47 (44)	61 (56)	1.01 (0.57, 1.78)
$20,000–49,999	118 (24)	49 (45)	60 (55)	1.14 (0.67, 1.94)
$50,000–74,999	75 (15)	24 (34)	47(66)	0.76 (0.42, 1.38)
≥$75,000	179 (37)	67 (38)	108 (62)	1.00 (ref)
***Employment Status***				
Not Working	256 (53)	103 (43)	139 (57)	1.25 (0.82, 1.92)
Working	230 (47)	84 (38)	137 (62)	1.00 (ref)
***Status of Marijuana Legalization in State of Residence***				
Recreational	197 (41)	75 (40)	112 (60)	1.08 (0.67, 1.75)
Medical	129 (27)	50 (40)	74 (60)	0.94 (0.57, 1.53)
Other	160 (33)	62 (41)	90 (59)	1.00 (ref)

*Numbers are unweighted, and percentages are weighted to approximate the US population. We used weights provided by GfK to approximate the US population based on socio-demographic factors (e.g., age, gender, race, ethnicity, education, household income, home ownership, and metropolitan area).

## Discussion

In a nationally representative survey of US adults, substitution of marijuana for opioids, which included a substantial degree of opioid discontinuation (~20%), was common. Better self-reported pain management and fewer side effects and withdrawal symptoms were the most common reasons for substitution. Our findings are consistent with prior surveys of American and Canadian marijuana users in which substitution of marijuana for opioids was prevalent due to better symptom management and fewer adverse and withdrawal effects [8].

Our study overcomes the potentially biased reporting in favor of substitution from prior convenience samples of marijuana users. This may explain why the prevalence of substitution in our study was lower than that of other studies in which a prevalence of up to 97% has been reported [8]. Additionally, we focused specifically on substitution of marijuana for opioids and asked about this practice directly whereas other studies asked about substitution of marijuana for prescription drugs more broadly [18–20] or indirectly assessed opioid substitution [21]. Our results were also inconsistent with a recently published Australian cohort study which followed approximately 1,500 people with chronic non-cancer pain prescribed opioids for four years [22]. About a third of participants reported that they sometimes or regularly reduced their opioid medication when using cannabis. However, the prevalence of opioid discontinuation was not significantly different between daily or near-daily marijuana users and non-users [22]. To date, data on self-reported improvement of symptoms has not been substantiated by studies that have monitored opioid and marijuana use. More research on this topic is clearly needed. Nonetheless, our findings suggest that even if objective measures do not support that marijuana is substitutive for opioid use, patients perceive that marijuana use has reduced their opioid use. Perhaps the commercialization of marijuana and the favorable media coverage surrounding the health effects of marijuana are fostering such a perception [23, 24]. More research including clinical trials on the efficacy of cannabis in pain management with the inclusion of patient-centered outcomes is needed to shed light on the role of marijuana on pain management.

Our study has several limitations that deserve comment. Our study was a cross-sectional survey of a relatively small number of respondents with a history of marijuana use and opioid use within the past year. Our survey question provided a limited number of examples of opioid medications (i.e., Vicodin, Percocet, or OxyContin), so it is possible that we did not identify all opioid users. Although our survey specifically asked about the use of opioids for pain, it is possible that we captured individuals who were using opioids for other reasons such as opioid use disorder. Likewise, it is possible that respondents may be using marijuana for reasons other than pain. Thus, we cannot conclude with certainty that patients are using marijuana as an alternative to opioids for pain per se. We also relied on respondents’ retrospective judgment regarding reduction or cessation of opioid use attributable to marijuana use. Thus, our findings could also be limited by recall bias, although this was minimized by restricting the sample to those who used opioids within the past year. Underreporting of substitution could have occurred, particularly in states in which marijuana has not been legalized.

## Conclusions

In conclusion, our study provides further evidence that patients self-report that they are substituting marijuana for opioids. The impact of marijuana substitution for opioids and concomitant opioid and marijuana use on health outcomes is unknown and warrants investigation.

## Supporting information

S1 File Tables A and B. Baseline Characteristics of Respondents Compared to NSDUH (US Adults 18 Years and Older) and Supplementary Questionnaire: Opiate Use Related Questions.
(DOCX)
