# Supplementary material for: Substitution of marijuana for opioids in a national survey of US adults
Source: PLoS One. 2019 Oct 4;14(10):e0222577. doi: 10.1371/journal.pone.0222577 (PMC6777788; doi:10.1371/journal.pone.0222577)
Supplement: S1 File — Tables A and B. Baseline Characteristics of Respondents Compared to NSDUH (US Adults 18 Years and Older) and Supplementary Questionnaire: Opiate Use Related Questions. (DOCX) [file pone.0222577.s001.docx]

**Table A in S1 File. Baseline Characteristics of Respondents Compared to NSDUH (US Adults 18 Years and Older)**

|  | **KN 2017**  **Weighted data**  **n = 9,003**  **n (%)** | **NSDUH 2015**  **Weighted data**  **n = 43,561**  **n (%)** |
| --- | --- | --- |
| ***Age (years)*** |  |  |
| 18-34 | 2543 (29) | 23637 (30) |
| 35-49 | 2172 (24) | 11164 (25) |
| 50-64 | 2466 (27) | 5157 (26) |
| ≥65 | 1822 (20) | 3598 (19) |
|  | | |
| ***Gender*** |  |  |
| Male | 4325 (48) | 19828 (48) |
| Female | 4678 (52) | 23733 (52) |
|  | | |
| ***Race*** |  |  |
| White/Non-Hispanic | 5772 (64) | 26025 (65) |
| Black/Non-Hispanic | 1067 (12) | 5502 (12) |
| Hispanic | 1430 (16) | 7648 (15) |
| Other/Non-Hispanic | 734 (8) | 4386 (8) |
|  | | |
| ***Education*** |  |  |
| High school or less | 3573 (39) | 18081 (40) |
| Some college | 2579 (29) | 14504 (30) |
| Bachelor’s degree or higher | 2850 (32) | 10976 (30) |
|  | | |
| ***Employment Status*** |  |  |
| Working | 5579 (62) | 29183 (62) |
| Not Working | 3424 (38) | 14378 (38) |
|  | | |
| ***Mean Household Size*** | 3 | 3^a^ |
|  | | |
| ***Household Income*** |  |  |
| <$20,000 | 1074 (12) | 9703 (18) |
| $20,000-49,999 | 2075 (23) | 14015 (30) |
| $50,000-74,999 | 1567 (17) | 6770 (17) |
| $75,000 or greater | 4287 (48) | 13073 (35) |
|  | | |
| ***Past Year Marijuana Use*** | 1314 (15) | 8292 (14) |

^a^Mean household size was calculated by considering respondents in the “6 or more category” as a value of 6

**Table B in S1 File. Supplementary Questionnaire: Opiate Use Related Questions***

| **Question** | **Responses** |
| --- | --- |
| 1. Have you ever used marijuana? | - Yes - No |
| 1. How long has it been since you last used marijuana? | - Within the past 30 days - More than 30 days but within the past 6 months - More than 6 months but within the past 12 months - More than 12 months ago |
| 1. In the past 12 months, have you regularly taken opiate medications such as Vicodin, Percocet, or OxyContin to treat pain? Do not include pain medications that can be bought without a prescription such as aspirin, Tylenol, or Advil. | - Yes - No |
| 1. In the past 12 months, how often have you used opiate pain medications on average? | - Daily - Weekly - Monthly - Less than monthly |
| 1. Have you noticed a change in the amount of opiate medications you need or use for pain because of your marijuana use? | - Yes, I need ***a lot more*** opiate medication - Yes, I need ***slightly more*** opiate medication - No change - Yes, I need ***slightly less*** opiate medication - Yes, I need ***a lot less*** opiate medication - I have been able to ***stop*** using opiate medications |
| *Need slightly less, a lot less, or have been able to stop using opiate medications:*  *Q.6*  *Need a lot more, slightly more, or no change in opiate medication: finished* | |
| 1. Why did you decrease or stop your opiate use? Check all that apply. | - Better pain management with marijuana - Fewer side effects from marijuana - Fewer withdrawal symptoms with marijuana - Marijuana is easier to obtain - Marijuana is cheaper - More social acceptance from marijuana use - Other: |

*Only questions relevant to this manuscript are included in this supporting material.
